# Brain Stem Encephalitis in a Patient With Recurrent Small Cell Lung Cancer Treated With Immune Checkpoint Inhibitor: Case Presentation and Review of the Literature

**DOI:** 10.7759/cureus.13034

**Published:** 2021-01-31

**Authors:** Noha N Soror, Lori Hemrock, Parth Shah, Richard J Loges, Biswaraj Tharu

**Affiliations:** 1 Internal Medicine, Western Reserve Health Education/Northeast Ohio Medical University (NEOMED), Warren, USA; 2 Medical Oncology, The Hope Center for Cancer Care, Warren, USA; 3 Radiology, Trumbull Regional Medical Center, Warren, USA; 4 Internal Medicine, Western Reserve Health Education/Northeast Ohio Medical University (NEOMED), Youngstown, USA

**Keywords:** small cell lung cancer(sclc), immune check-point inhibitor, immune check point inhibition, immune-related adverse effects, brain stem encephalitis, immuo-oncology

## Abstract

Immunotherapy with checkpoint inhibitors (CPIs) has revolutionized the management of advanced cancer including advanced small cell lung cancer (SCLC). Unfortunately, those agents are not without adverse effects. Immune imbalance through enhanced cellular immune response may result in impaired endogenous immunological tolerance mechanisms that can result in a wide spectrum of immunological side effects also known as immune-related adverse events (irAEs). Scarce data are currently available about neurological immune-related adverse events (neuro irAEs), mainly obtained from clinical trials, case reports, or small case series. Most reported cases presented with nonspecific symptoms. It is important to recognize and promptly treat neuro irAEs, as it may be serious and even potentially fatal.

We present a rare case of nivolumab induced brain stem encephalitis in a patient with advanced SCLC presented 10 months after starting treatment with symptoms of nystagmus, gait disturbance, and blurry vision. Nivolumab was held and the patient was started on oral steroids with tapering dose. The patient’s symptoms gradually improved over a few weeks. Re-challenging with nivolumab six weeks later resulted in recurrence of symptoms and again the patient was prescribed oral steroids with tapering dose. She maintained response off treatment for six months. This case report is aimed to highlight the importance of clinically suspecting and promptly treating neurological irAE, when managing a patient with CPIs.

## Introduction

Checkpoint inhibitors (CPIs) are monoclonal antibodies targeting cytotoxic T-lymphocyte-associated antigen 4 (CTLA-4), programmed death 1 (PD-1), or programmed death-ligand 1 (PD-L1). Those agents demonstrated remarkable efficacy and survival benefit in different advanced malignancies including advanced small cell lung cancer (SCLC) [1]. Unfortunately, CPI can cause immune imbalance that can manifest as a wide range of immunological side effects known as immune-related adverse effects (irAEs). IrAEs include various dermatological, endocrine, gastrointestinal/hepatic and sometimes inflammatory events [2]. Here we report an unusual case of nivolumab-induced brain stem encephalitis in a patient with recurrent SCLC.

## Case presentation

A 66-year-old female presented with a history of SCLC of right middle lung lobe (stage 1). She has undergone right middle lobe (RML) lobectomy followed by four cycles of adjuvant carboplatin and etoposide. Fourteen month later, she developed recurrence in her subcarinal/paratracheal nodes confirmed on endobronchial ultrasound (EBUS) biopsy. She then had concurrent chemotherapy with radiation completed followed by prophylactic cranial irradiation. Again she developed recurrent disease in celiac axis nodes confirmed on biopsy for which she was started on nivolumab. Ten months after starting nivolumab therapy, the patient presented to the office complaining of dizziness, imbalance, and double vision. Past medical history included chronic obstructive pulmonary disease (COPD), diabetes mellitus type 2, neuropathy, and myocardial infarction (MI). She was a former smoker with 75 PPD smoking history and had a family history significant of lung cancer. 

**Figure 1:**
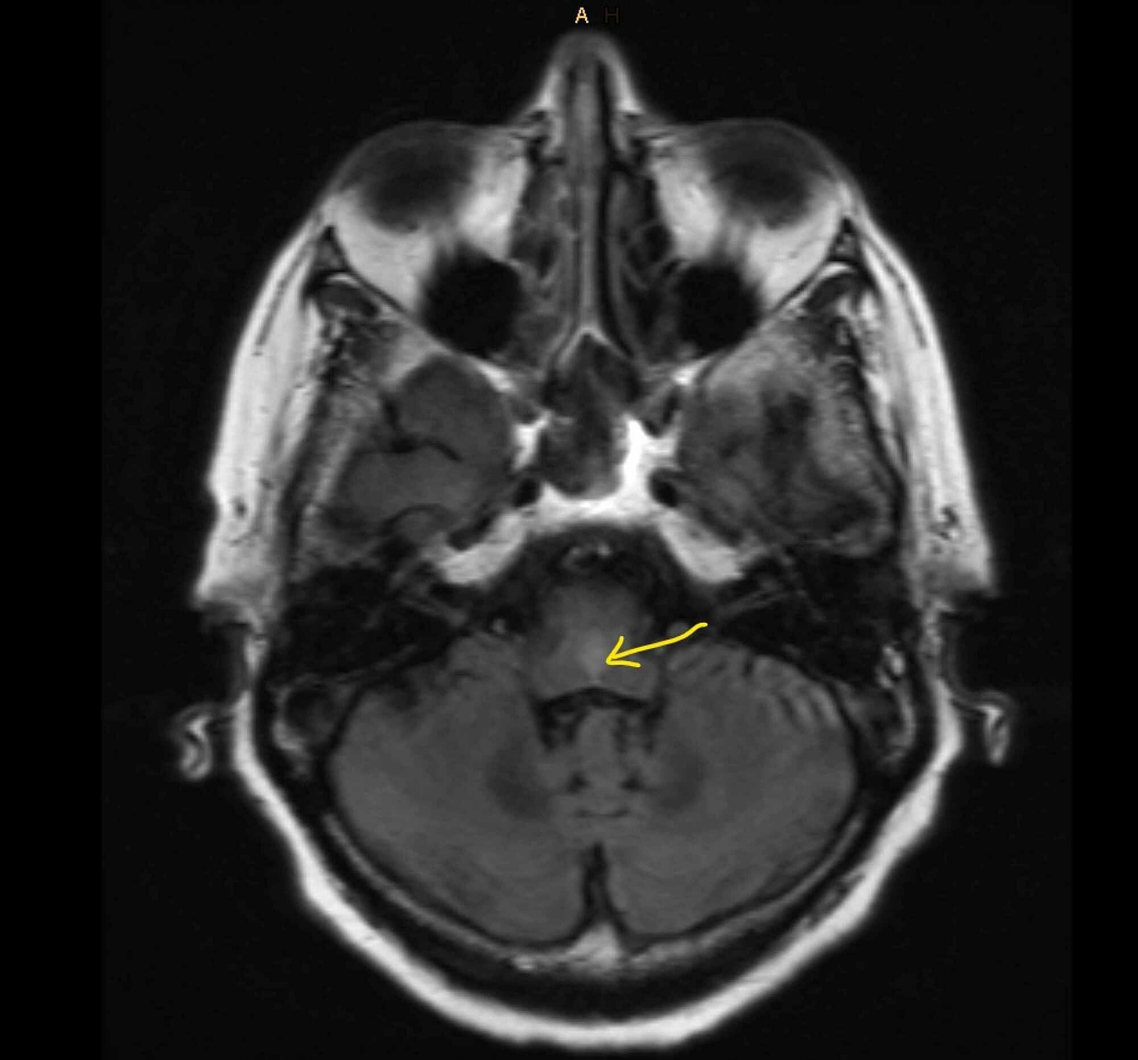
T2 FLAIR before treatment. FLAIR, fluid-attenuated inversion recovery

Physical examination was positive for a significant vertical nystagmus. MRI of the brain in November, 2018 showed extensive symmetric linear hyperintensity in T2 Flair throughout medulla, dorsal pons, and midbrain with post contrast enhancement (Figure 1).

**Figure 2:**
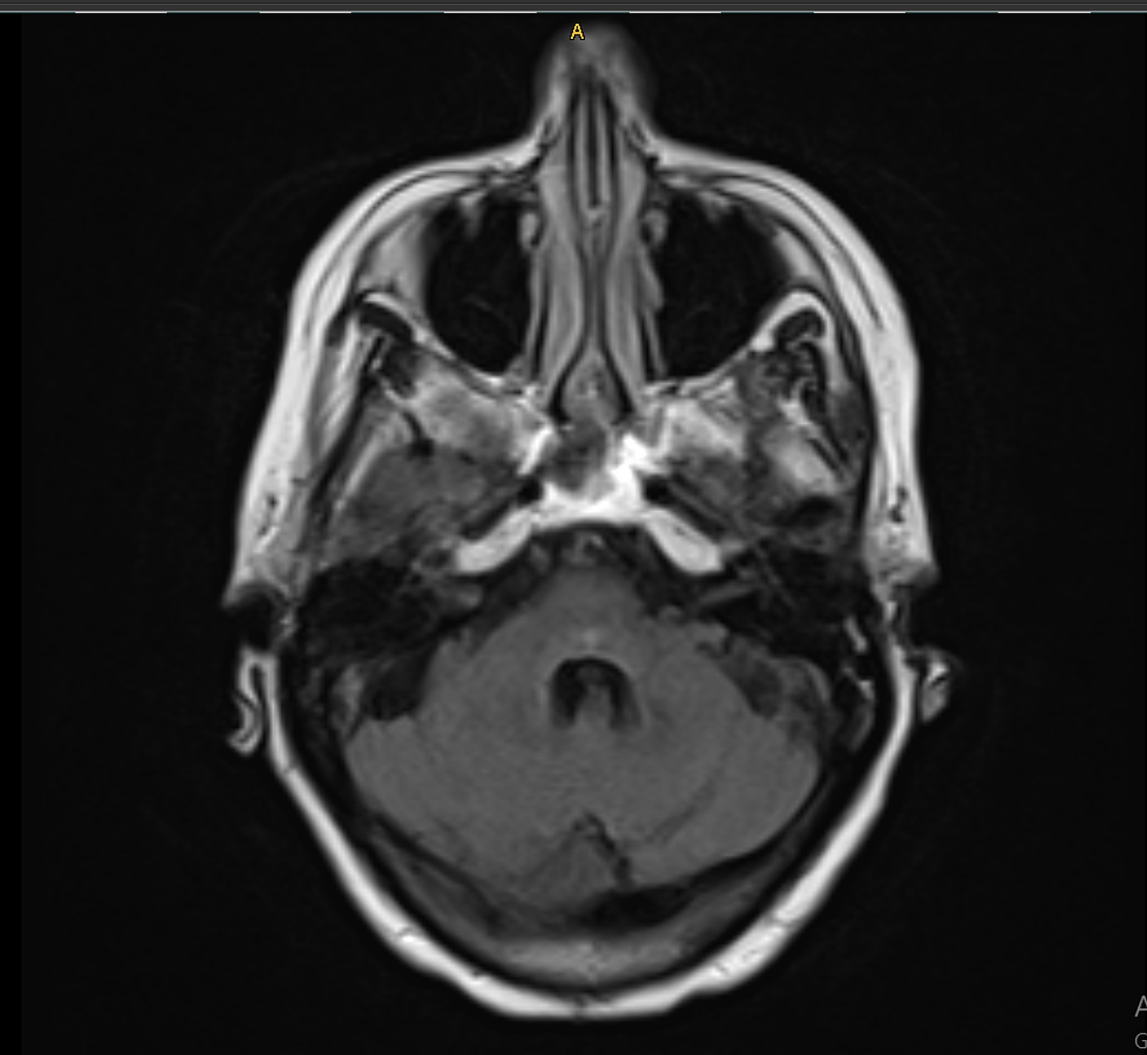
T2 FLAIR post-treatment. FLAIR, fluid-attenuated inversion recovery

The patient was started on oral steroids with a tapering dose. Nivolumab was held for six weeks and her symptoms improved over this period. Then she was given a re-challenge dose of nivolumab but her vision and balance worsened again. Again, she was started on oral steroids with a tapering dose and nivolumab was held. Blurry vision and imbalance were improved within a few days, but nystagmus gradually improved over five months. The patient remained off treatment with observation alone for nine months when MRI of the brain showed resolution of abnormal linear enhancement of the brain parenchyma (Figure 2), however, follow up CT demonstrated focal area of consolidation in superior lobe of right lung and enlarged posterior pancreatic lymph node.

The positron emission tomography (PET) scan showed mild activity nodule in right upper lobe (RUL), pleural-based suspicious soft tissue mass, and PET positive nodule in peri-portal region. She was started on pembrolizumab and has had six doses so far. Most recent follow up PET/CT August 2020 showed no fluorodeoxyglucose (FDG) avid uptake.

## Discussion

Checkpoint inhibitors are immunomodulatory antibodies that have been successfully used to enhance the immune system in patients with advanced cancer. They are approved for treatment of different advanced malignancies [1]. This led to improved prognosis and survival in patients with different advanced malignancies. Unfortunately, general immunological enhancement can lead to a spectrum of adverse effects known as irAEs. Treatment of irAEs mandates interruption or sometimes discontinuation of the CPI and temporary immunosuppression with corticosteroids, or other immunosuppressive agents [2].

Patients with moderate immune-mediated toxicities (grade 2); withholding immunotherapy should be the first step. Corticosteroids can be started if symptoms do not resolve within a week, While, for patients with severe or life-threatening (grades 3 and 4) irAEs, CPIs should be indefinitely discontinued and high dose steroids should be started. Tapering steroids can then be initiated once symptoms subside [3].

Nivolumab is an antibody against PD-1 that is approved for treating various types of advanced cancers including SCLC. IrAEs are significantly less frequent with the anti-programmed cell death receptor 1 (PD-1) antibodies compared with cytotoxic T-lymphocyte-associated antigen 4 (CTLA-4). One pooled analysis reporting nAE has reported that nivolumab-treated patients had higher incidence of nAEs as compared to patients who received ipilimumab or pembrolizumab (7% vs. 1% and 2%) and that combination therapy harbored the highest incidence of developing nAEs [4].

Dermatological, gastrointestinal (GI), and endocrine toxicities are the most common irAEs associated with CPIs. Neurotoxicity occurs in approximately 1%-14% of patients [5-6]. Autoimmune encephalitis is extremely rare but serious and sometimes potentially fatal irAE. Neurological irAEs typically develop within months of starting the therapy [7].

Reintroducing CPI also known as re-challenging after development of severe irAEs can be tried by either, class switch from anti-PD-(L)1 to anti-CTLA-4 therapy or vice versa in diseases where both classes are appropriate treatment option; or by re-challenge scenario with the reintroduction of the same class agent or the same molecule after resolution of the irAE [8]. Recent study found that re-challenge with the same immune CPI after a specific irAE would result in 28.8% recurrence rate of the same irAE associated with the discontinuation of ICI therapy [9]. Early recognition and intervention are crucial for reducing severity and impact of toxicity [10].

## Conclusions

This patient suffered nivolumab-induced brain stem encephalitis that resulted in nystagmus, imbalance, and acute vision changes. Steroid therapy was effective for treating vision changes and nystagmus. Unfortunately, re-challenge with nivolumab resulted in recurrence of symptoms. Switching to another CPI; pemprolizumab did not result in recurrence of symptoms. Early recognition and prompt treatment of neurological irAE might be lifesaving. 
